# Hilar Cholangiocarcinoma with Para-Aortic Lymph Node Metastasis Treated with Chemoimmunotherapy and Conversion Surgery: A Case Report

**DOI:** 10.70352/scrj.cr.25-0023

**Published:** 2025-03-19

**Authors:** Yuma Yasui, Koichi Kimura, Norifumi Iseda, Yoshinari Nobuto, Hiroko Yano, Yuichiro Kajiwara, Takuro Watanabe, Fang Cao, Michiko Amano, Takaaki Tanaka, Hironori Ochi, Nobuaki Azemoto, Kazuhito Minami, Ryosuke Minagawa, Tomoyuki Yokota, Takashi Nishizaki

**Affiliations:** 1 Department of Surgery, Matsuyama Red Cross Hospital, Matsuyama, Ehime, Japan; 2 Center for Liver-Biliary-Pancreatic Disease, Matsuyama Red Cross Hospital, Matsuyama, Ehime, Japan

**Keywords:** cholangiocarcinoma, durvalumab, conversion surgery, complete response

## Abstract

**INTRODUCTION:**

Cholangiocarcinoma (CC) has a poor prognosis and few treatment options. Conversion surgery for unresectable CC has been frequently reported; however, there are almost no reports of conversion surgery after durvalumab plus gemcitabine and cisplatin therapy. In this study, we report the case of a patient with unresectable hilar CC who received durvalumab plus gemcitabine and cisplatin therapy and achieved a pathological complete response after conversion surgery.

**CASE PRESENTATION:**

A 70-year-old man was diagnosed with hilar CC (cT3N1M0, Stage III C) based on biopsy of the common bile duct stenosis and computed tomography (CT) and magnetic resonance cholangiopancreatography scans. Initially, a right lobe hepatectomy and subtotal stomach-preserving pancreatoduodenectomy were planned. However, there were concerns about an insufficient functional remnant liver volume. Trans-ileocolic portal embolization of the right portal vein branch was performed. On a preoperative CT scan 1 month later for liver volumetry, swelling of the para-aortic lymph nodes was observed, which was judged as distant metastasis, and radical resection could not be performed. After 8 courses of durvalumab plus gemcitabine and cisplatin therapy, vanishing fluorodeoxyglucose accumulation in the para-aortic lymph nodes was observed on positron emission tomography-CT. The possibility of resection was reevaluated, and a right lobe hepatectomy and extrahepatic biliary reconstruction were performed as conversion surgeries. Histological examination confirmed the absence of residual tumors or lymph node metastases. Ten months after surgery, the patient was free of recurrence.

**CONCLUSIONS:**

Chemoimmunotherapy with durvalumab as a first-line treatment for unresectable CC has shown promising results. Immunotherapy with durvalumab, followed by conversion surgery, may improve the prognosis of patients with unresectable CC.

## Abbreviations

CC
: cholangiocarcinoma
CT
: computed tomography
pCR
: pathological complete response
RLV
: remnant liver volume
SLV
: standard liver volume
TLV
: total liver volume

## INTRODUCTION

Cholangiocarcinoma (CC) has a poor prognosis and few treatment options. Surgical resection is the only curative treatment for CC.^1)^ However, in many cases, CC is already an advanced cancer that is difficult to resect radically by the time clinical symptoms appear.^2)^ In cases of unresectable or metastatic CC, gemcitabine-based chemotherapy may be a treatment option.^3,4)^ In recent years, the addition of immune checkpoint inhibitors to chemotherapy has been shown to improve overall survival and objective response rates, and is recommended as a treatment for unresectable CC.^5,6)^

Conversion surgery after chemotherapy for liver cancer, pancreatic cancer, and CC was first reported by Bismuth et al. in 1996.^7)^ Currently, an increasing number of reports suggest that conversion surgery after chemotherapy can improve the prognosis for various types of cancer.^8–10)^ In the field of CC, the number of patients with initially unresectable CC who underwent resection as a conversion surgery has been increasing.^11)^ Nevertheless, there are few reports on the use of immunochemotherapy and conversion surgery for unresectable CC, and there are few reports on achieving a pathological complete response (pCR).^12)^ In this study, we report the case of a patient with unresectable hilar CC who received durvalumab plus gemcitabine and cisplatin therapy and achieved a pCR after conversion surgery.

## CASE PRESENTATION

**Fig. 1:**
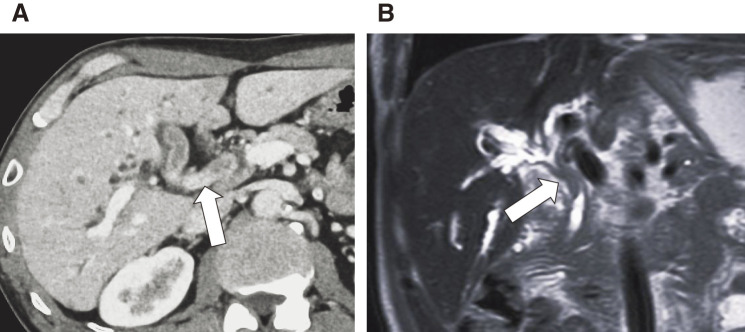
Initial computed tomography (A) and magnetic resonance imaging (B) scans at the time of diagnosis. The images demonstrate a soft tissue mass in the common bile duct at the level of the confluence of the choledochal ducts (white arrows).

A 70-year-old man was referred to our hospital after a jaundice was reported by another physician. Blood examination revealed elevated hepatobiliary enzymes and increased levels of tumor markers (CA19-9, 444.3 U/mL; carcinoembryonic antigen (CEA), 15.5 ng/mL). Contrast-enhanced computed tomography (CT) and magnetic resonance cholangiopancreatography revealed Bismuth classification II^13)^ bile duct wall thickening from the confluence of the right and left hepatic ducts to the confluence of the choledochal ducts, with intrahepatic bile duct dilation. The tumor had partially reached the intrapancreatic bile duct. Hilar lymph node enlargement was observed, and lymph node metastasis was suspected. No distant metastases were observed (**Figs. 1A** and **1B**). A biopsy of the common bile duct stenosis revealed adenocarcinoma, and the patient was diagnosed with hilar CC (cT3N1M0, Stage III C). Subtotal stomach-preserving pancreatoduodenectomy with right lobe hepatectomy was planned as radical resection. CT volumetry revealed the following: total liver volume (TLV) = 1350 mL, standard liver volume (SLV) = 1118.2 mL, remnant liver volume (RLV) = 483 mL, RLV/TLV = 35.8%, and RLV/SLV = 43.2%. Trans-ileocolic right portal vein embolization was performed^14)^ because of concerns about an insufficient remaining liver volume.

**Fig. 2:**
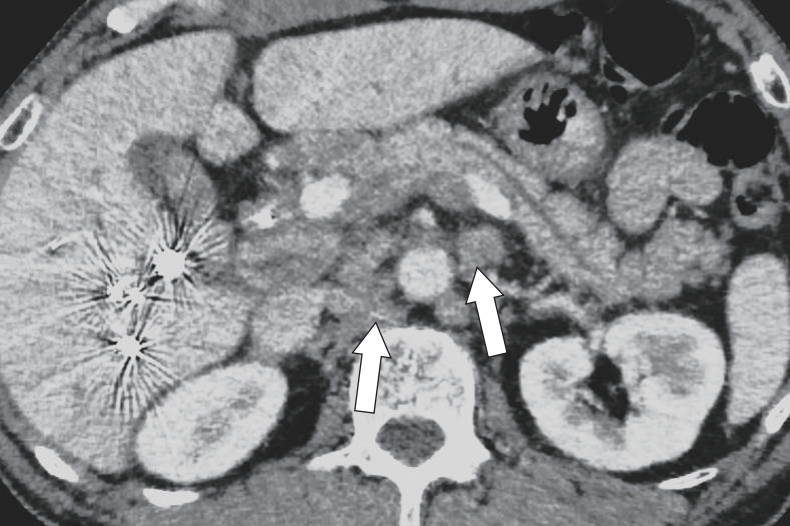
Interval computed tomography scan after right portal vein embolization. The image demonstrates para-aortic lymph node enlargement (white arrows).

However, a CT performed 1 month after trans-ileocolic right portal vein embolization revealed enlarged para-aortic lymph nodes that were considered metastatic, and radical resection was judged to be unsuitable (**Fig. 2**). Therefore, the decision was made to administer systemic chemotherapy. Following the treatment protocol of the TOPAZ-1 study,^5)^ cisplatin (25 mg/m^2^) and gemcitabine (1000 mg/m^2^) were administered on days 1 and 8 of each 21-day cycle for 8 cycles, and durvalumab (1500 mg) was administered on day 1 of each cycle. The patient was followed up with monthly tumor marker measurements and CT scans every 3 months. At the end of the 4th course, the CA19-9 and CEA levels decreased to the normal range. Positron emission tomography-CT revealed vanishing fluorodeoxyglucose accumulation in the para-aortic lymph nodes. Systemic chemotherapy was deemed to be significantly effective, and radical resection was considered. Then, when the chemotherapy response was confirmed, the possibility of surgery was explained to the patient. The patient strongly preferred radical surgery over continued chemotherapy, and the decision was made to proceed with radical surgery.

**Fig. 3:**
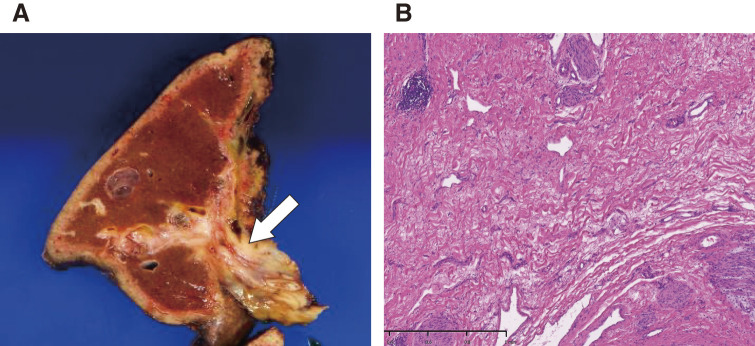
(A) Clinical photograph of the resected specimen. A well-defined sclerotic lesion is seen along the right hepatic duct (white arrow). (B) Hematoxylin–eosin staining of liver tumor tissue from the resected specimen. Only fibrosis, without malignancy, is observed.

Second CT volumetry before surgery showed the following: TLV = 1231 mL, SLV = 1167.6 mL, RLV = 745 mL, RVL/TLV = 60.5%, and RLV/SLV = 63.8%. Endoscopic retrograde cholangiopancreatography biopsies of the original left hepatic duct and the cephalic portion of the intrapancreatic bile duct were negative for malignancy. An open right lobe hepatectomy and extrahepatic biliary reconstruction were also performed. The operative time was 10 h 40 min, blood loss was 460 g, and the weight of the resected liver was 474 g. Visually, a well-defined white sclerotic lesion proliferating along the right hepatic duct was observed (**Fig. 3A**). Histologically, there was no evidence of carcinoma, fibrous stroma-rich fibrotic scarring associated with treatment, or lymph node metastasis (**Fig. 3B**). The final TNM classification (UICC-8th) was ypT0N0. One month after surgery, a Grade 3 skin disorder appeared, and the patient was treated by the dermatology department. The patient showed no recurrence 10 months after surgery.

## DISCUSSION

pCR in unresectable CC is infrequent; only a few case reports have described the use of durvalumab plus gemcitabine and cisplatin therapy followed by conversion surgery to achieve pCR.^15,16)^ This is the first report of a patient with unresectable hilar CC who underwent conversion surgery after durvalumab plus gemcitabine and cisplatin therapy and achieved a pCR.

The ABC-02 study established gemcitabine/cisplatin therapy as the international standard treatment for unresectable CC.^4)^ Since then, cytotoxic triplet therapy, molecular-targeted drugs, and immunotherapy have been investigated, and gemcitabine/cisplatin/S-1, gemcitabine/cisplatin/durvalumab, and gemcitabine/cisplatin/pembrolizumab have been shown to be beneficial. In a phase III randomized controlled trial, the objective response rates for gemcitabine/cisplatin/S-1, gemcitabine/cisplatin/durvalumab, and gemcitabine/cisplatin/pembrolizumab were 41.5%, 26.7%, and 29.0%, respectively.^5,6,17,18)^ Although there are no reports comparing these drugs directly, we speculate that gemcitabine/cisplatin/S-1 is a better choice in terms of objective response for performing conversion surgery.

The expression of programmed death ligand 1, DNA damage repair pathway-related gene mutations, and the tumor microenvironment have been reported as predictors of immunochemotherapy in patients with CC, but the findings obtained are limited.^19,20)^ The TOPAZ-1 study concluded that the addition of durvalumab immunochemotherapy to standard gemcitabine/cisplatin therapy improved the objective response; this benefit was independent of programmed death ligand 1 expression and was observed in all population subtypes.^5)^ In non-small cell lung cancer and head and neck cancer, the administration of immune checkpoint inhibitors has been reported to change the cancer immune microenvironment and increase tumor sensitivity to chemotherapy.^21,22)^ In the present case, the patient chose to receive durvalumab combination therapy to enhance sensitivity to gemcitabine/cisplatin chemotherapy and retain S-1 as a second-line chemotherapy option. The durvalumab combination therapy was effective and enabled conversion surgery. Investigating the genomic mutation profile in this case may help elucidate the prognostic factors for immunochemotherapy in patients with advanced CC.

Whether it is better to continue chemotherapy after chemotherapy for unresectable CC or to switch to conversion surgery remains debatable. In this case, the patient had a strong desire for radical surgery, and conversion surgery was performed. Kato et al. reported that the median survival time for 10 patients with unresectable locally advanced CC who underwent conversion surgery was 17.9 months.^11)^ Yabushita et al. reported that the median survival time of 56 patients with locally advanced unresectable CC treated with conversion surgery was 37.7 months, the 5-year survival rate was 39.1%, and 43 patients underwent R0 resection.^23)^ These reports suggest that preoperative downsizing chemotherapy prolongs survival after conversion surgery in patients with initially unresectable locally advanced CC. Leong et al. reported a case of locally advanced gallbladder cancer that was treated with durvalumab plus gemcitabine and cisplatin, followed by conversion surgery, and was subsequently R0-resected, with no recurrence for 6 months.^15)^ In this case, the patient received gemcitabine/cisplatin/durvalumab chemotherapy for unresectable CC and showed a remarkable response to treatment. After surgical resection, the patient achieved a pCR and remained relapse-free for 10 months. For unresectable CC, achieving R0 resection through conversion surgery after the initiation of chemotherapy may prolong the prognosis. However, there are no firm data on the optimal timing of surgery after systemic chemotherapy for unresectable CC.^24)^

## CONCLUSIONS

In conclusion, we encountered a valuable case of unresectable hilar CC in a patient who underwent conversion surgery after durvalumab plus gemcitabine and cisplatin therapy and achieved a pCR. Chemotherapy with durvalumab shows promising results as a first-line treatment for unresectable CC. Immunotherapy with durvalumab, followed by conversion surgery, may further improve the prognosis of patients with unresectable CC.

## ACKNOWLEDGMENTS

We would like to thank Editage (http://www.editage.jp/) for English language editing of this manuscript.

## DECLARATIONS

### Funding

This study received no specific grants from any funding agency in the public, commercial, or not-for-profit sectors.

### Authors’ contributions

YY collected the associated data and edited the manuscript.

KK, RM, KM, TY, and TN supervised manuscript writing.

NI, YN, HY, YK, TW, FC, MA, TT, HO, and NA participated in treatment.

All the authors have read and approved the final version of the manuscript.

### Availability of data and materials

Not applicable.

### Ethics approval and consent to participate

Not applicable.

### Consent for publication

Written informed consent was obtained from the patient for publication of this case report and accompanying images.

### Competing interests

The authors declare that they have no competing interests.

## References

[1] Valle JW Kelley RK Nervi B Biliary tract cancer. Lancet 2021; 397: 428–44. 33516341 10.1016/S0140-6736(21)00153-7

[2] Khan SA Davidson BR Goldin RD Guidelines for the diagnosis and treatment of cholangiocarcinoma: an update. Gut 2012; 61: 1657–69. 22895392 10.1136/gutjnl-2011-301748

[3] Okusaka T Ishii H Funakoshi A Phase II study of single-agent gemcitabine in patients with advanced biliary tract cancer. Cancer Chemother Pharmacol 2006; 57: 647–53. 16142487 10.1007/s00280-005-0095-3

[4] Valle J Wasan H Palmer DH Cisplatin plus gemcitabine versus gemcitabine for biliary tract cancer. N Engl J Med 2010; 362: 1273–81. 20375404 10.1056/NEJMoa0908721

[5] Oh D-Y Ruth He A Qin S Durvalumab plus gemcitabine and cisplatin in advanced biliary tract cancer. NEJM Evid 2022; 1: EVIDoa2200015. 38319896 10.1056/EVIDoa2200015

[6] Kelley RK Ueno M Yoo C Pembrolizumab in combination with gemcitabine and cisplatin compared with gemcitabine and cisplatin alone for patients with advanced biliary tract cancer (KEYNOTE-966): a randomised, double-blind, placebo-controlled, phase 3 trial. Lancet 2023; 401: 1853–65. 37075781 10.1016/S0140-6736(23)00727-4

[7] Bismuth H Adam R Lévi F Resection of nonresectable liver metastases from colorectal cancer after neoadjuvant chemotherapy. Ann Surg 1996; 224: 509–20; discussion 520–2. 8857855 10.1097/00000658-199610000-00009 PMC1235414

[8] Shoji Y Kanamori K Koyanagi K Conversion surgery for esophageal and esophagogastric junction cancer. Int J Clin Oncol 2024; 29: 1777–84. 39436571 10.1007/s10147-024-02639-4 PMC11588808

[9] Deng H Liu J Cai X Radical minimally invasive surgery after immuno-chemotherapy in Initially-unresectable Stage IIIB non-small cell Lung Cancer. Ann Surg 2022; 275: e600–2. 34596079 10.1097/SLA.0000000000005233

[10] Iseda N Itoh S Toshima T Outcome of hepatectomy after systemic therapy for hepatocellular carcinoma: a Japanese multicenter study. Surg Today 2024. [Online ahead of print] 10.1007/s00595-024-02930-x 39192138

[11] Kato A Shimizu H Ohtsuka M Downsizing chemotherapy for initially unresectable locally advanced biliary tract cancer patients treated with gemcitabine plus cisplatin combination therapy followed by radical surgery. Ann Surg Oncol 2015; 22 (Suppl 3): S1093–9. 26240009 10.1245/s10434-015-4768-9

[12] Wang S Wang Y Zhu C Conversion surgery intervention versus continued systemic therapy in patients with a response after PD-1/PD-L1 inhibitor-based combination therapy for initially unresectable biliary tract cancer: a retrospective cohort study. Int J Surg 2024; 110: 4608–16. 38704621 10.1097/JS9.0000000000001540 PMC11326034

[13] Bismuth H Castaing D Traynor O. Resection or palliation: priority of surgery in the treatment of hilar cancer. World J Surg 1988; 12: 39–47. 2449769 10.1007/BF01658484

[14] Kimura K Minagawa R Yamaoka T Transileocolic portal vein embolization increases remnant liver volume after major hepatectomy. In Vivo 2024; 38: 2761–6. 39477424 10.21873/invivo.13755 PMC11535930

[15] Leong EKF Tan NCH Pang NQ Case report: from palliative to potentially curative – the advent of immunotherapy providing hope to advanced gallbladder adenocarcinoma. Front Immunol 2024; 15: 1353430. 38370411 10.3389/fimmu.2024.1353430 PMC10869450

[16] Orlandi E Toscani I Trubini S Evolving approaches in advanced gallbladder cancer with complete pathological response using chemo-immunotherapy: a case report. Oncol Lett 2024; 28: 473. 39161332 10.3892/ol.2024.14606 PMC11332581

[17] Ioka T Kanai M Kobayashi S Randomized phase III study of gemcitabine, cisplatin plus S-1 versus gemcitabine, cisplatin for advanced biliary tract cancer (KHBO1401- Mitsuba). J Hepatobiliary Pancreat Sci 2023; 30: 102–10. 35900311 10.1002/jhbp.1219 PMC10086809

[18] Merters J Lamarca A. Integrating cytotoxic, targeted and immune therapies for cholangiocarcinoma. J Hepatol 2023; 78: 652–7. 36400328 10.1016/j.jhep.2022.11.005

[19] Chen X Wang D Liu J Genomic alterations in biliary tract cancer predict prognosis and immunotherapy outcomes. J Immunother Cancer 2021; 9: e003214. 34795005 10.1136/jitc-2021-003214 PMC8603283

[20] Jian Z Fan J Shi GM Gemox chemotherapy in combination with anti-PD1 antibody toripalimab and lenvatinib as first-line treatment for advanced intrahepatic cholangiocarcinoma: a phase 2 clinical trial. J Clin Oncol 2021; 39 (15_suppl): 4094.

[21] Schvartsman G Peng SA Bis G Response rates to single-agent chemotherapy after exposure to immune checkpoint inhibitors in advanced non-small cell lung cancer. Lung Cancer 2017; 112: 90–5. 29191606 10.1016/j.lungcan.2017.07.034

[22] Saleh K Daste A Martin N Response to salvage chemotherapy after progression on immune checkpoint inhibitors in patients with recurrent and/or metastatic squamous cell carcinoma of the head and neck. Eur J Cancer 2019; 121: 123–9. 31574417 10.1016/j.ejca.2019.08.026

[23] Yabushita Y Park JS Yoon YS Conversion surgery for initially unresectable locally advanced biliary tract cancer: a multicenter collaborative study conducted in Japan and Korea. J Hepatobiliary Pancreat Sci 2024; 31: 481–91. 38822227 10.1002/jhbp.1437

[24] Noji T Nagayama M Imai K Conversion surgery for initially unresectable biliary malignancies: a multicenter retrospective cohort study. Surg Today 2020; 50: 1409–17. 32468112 10.1007/s00595-020-02031-5

